# The Role of Key Molecules of Pyroptosis in Liver Damage of Rats With Exertional Heat Stroke

**DOI:** 10.1155/grp/6864091

**Published:** 2025-01-20

**Authors:** Lifang Lin, Jiaolong Zheng, Qingqing Lin, Fangze Cai, Dongliang Li

**Affiliations:** ^1^Department of Hepatobiliary Disease, 900th Hospital of Joint Logistics Support Force, Fuzhou General Clinical Medical College of Fujian Medical University, Fuzhou, Fujian, China; ^2^Department of Gastroenterology, Fuzhou University Affiliated Provincial Hospital, Fuzhou, Fujian, China

## Abstract

**Purpose:** This study is aimed at investigating the role of key molecular elements involved in pyroptosis in liver injury caused by exertional heat stroke (EHS).

**Methods:** We established a model of EHS-induced liver injury in Sprague–Dawley rats, with a control group (receiving no treatment) for comparison and 12 rats in each group. Alanine transaminase (ALT) and aspartate transaminase (AST) levels in the blood were detected. Interleukin-1 beta (IL-1*β*) and interleukin-18 (IL-18) levels were assessed using enzyme-linked immunosorbent assays (ELISA). Pathological changes in liver tissue were examined by hematoxylin and eosin (H&E) staining. Quantitative real-time polymerase chain reaction (qRT-PCR) and Western blotting were used to detect mRNA and protein expression levels of Caspase-1 and Gasdermin D.

**Results:** Compared to the control group, the liver tissue of the EHS group showed congestion in hepatic sinusoids, hepatocyte edema, eosinophilic changes, necrosis, and infiltration of inflammatory cells. ALT and AST levels in the EHS group were significantly higher than those in the control group (*p* < 0.05). The mRNA expressions of Caspase-1, Gasdermin D, IL-1*β*, and IL-18 were significantly increased in the EHS group compared to the control group (*p* < 0.001). The protein expressions of Caspase-1, cleaved Caspase-1, Gasdermin D, and cleaved Gasdermin D were significantly increased in the EHS group.

**Conclusion:** These findings indicated that hepatic pyroptosis plays an important role in EHS-induced liver injury.

## 1. Introduction

Heat-related illness is a clinical syndrome characterized by a series of related clinical manifestations that occurs when the body's heat production capacity exceeds its heat dissipation capacity under conditions of high temperature and humidity. This leads to an increase in core body temperature, resulting in damage to tissues and organs. Heat stroke is the most severe form of heat stress–related illness, with a high fatality rate. It can be divided into classic heat stroke (CHS) and exertional heat stroke (EHS) according to the differences in the causes of the disease and the vulnerable population [1]. CHS mainly occurs in the elderly, immunocompromised individuals, or those with complications. EHS mostly occurs in young adults following strenuous exercise or physical labor. Animal studies showed that the thermal latency in EHS was shorter than that in CHS, and rhabdomyolysis and other forms of organ damage occurred earlier in EHS [2, 3]. In recent years, due to climate change, prolonged periods of high temperature during summer have become more common, with extreme weather events exceeding 39°C continuously occurring in some regions of the country. However, in the southeastern and southern coastal regions, where both high temperatures and humidity prevail during summer, the risk of developing EHS is increased.

The liver is one of the most vulnerable organs of EHS. Severe patients of EHS often suffer from liver failure. Studies have shown that the severity of liver injury in EHS patients is closely related to prognosis [4]. However, the precise mechanism of liver injury in EHS has not yet been fully elucidated. Previously, the pathogenesis of EHS was mainly focused on ischemia–reperfusion injury, endotoxemia, and apoptosis. However, recent studies have found that the increased levels of inflammatory factors, such as interleukin-1 beta (IL-1*β*) and interleukin-18 (IL-18), can be detected in vivo in the early stage of EHS, and these inflammatory factors cannot be reduced by inhibiting the activity of Caspase-3. Therefore, apoptosis is not the main form of cell injury in EHS pathophysiology. The role of pyroptosis in the pathogenesis of EHS has been reported recently. Pei, Geng, and Su found that heat stress can induce pyroptosis in umbilical vein endothelial cells, and this process can be significantly inhibited by Gasdermin D (GSDMD) siRNA [5]. Geng et al. also showed that heat stress induces pyroptosis in hepatocytes both in vivo and in vitro and that NLRP3-dependent pyroptosis contributes to liver injury after heat stress, which is one of the few known mechanisms [6]. Pyroptosis is a recently discovered form of programmed cell death, and it is characterized by the formation of pores in the cell membrane following the activation of GSDMD (cleaved GSDMD) by cleaved Caspase-1. This process leads to the release of intracellular inflammatory mediators and intracellular contents [7]. Consequently, the imbalance of ion concentration and osmotic pressure between intracellular and extracellular of the cell results in cell swelling, rupture, and death. The release of inflammatory mediators promotes the aggregation of inflammatory cells and further aggravates the inflammatory response [8]. Studies have found that pyroptosis plays an important role in septic liver injury. Multiple organ dysfunction (MOD) is common in EHS, a process similar to sepsis and sepsis-induced MOD [9], and pyroptosis plays an important role in septic liver injury. Existing studies on pyroptosis in EHS mostly focus on inflammasomes, while other key downstream molecules of the inflammasome, such as Caspase-1 and GSDMD, are still poorly understood.

Therefore, in this study, rat models with liver injury induced by EHS were established through exposure to high-temperature and high-humidity environment. The expression levels of Caspase-1 and GSDMD in the pyroptosis pathway as well as the levels of downstream inflammatory factors IL-1*β* and IL-18 were detected, to further reveal the pathogenesis of EHS. It provides a theoretical foundation for the prevention and treatment of EHS.

## 2. Materials and Methods

### 2.1. Experimental Rats

We purchased 30 clean-grade Sprague–Dawley male rats, aged 5–7 weeks and weighing 120–150 g, from Shanghai Slack Laboratory Animal Co. Ltd. (license number SCXK (Shanghai) 2017-0005). The animals were housed in individual cages at the animal experiment center of the 900th Hospital of the Joint Logistics Support Force of the Chinese People's Liberation Army, with five animals per cage. The animals were housed at a room temperature of 25°C ± 2°C and humidity of 60% ± 5%. The study was approved by the ethics committee of the 900th Hospital of the People's Liberation Army Joint Service Support Force.

### 2.2. Experimental Design

All 30 rats underwent a 7-day adaptive running training with progressively increasing load. During the training, the room temperature was maintained at 25°C ± 2°C and the humidity was 60% ± 5%. By Day 7, the running speed was 29 m/min and the training time was 25 min. Twenty four Sprague–Dawley rats that successfully completed the running were selected and randomly divided into two groups, with 12 rats in each group. In the EHS group (EHSG), the rats ran on the treadmill with a speed of 29 m/s under the environment temperature of 35°C ± 2°C and humidity of 80% ± 10%. After the core body temperature reached the diagnostic criteria of EHS, running was stopped, while the control group (CG) did not undergo any running.

### 2.3. Automatic Biochemical Analyzer to Detect Liver Function

The blood samples were collected by gently piercing the rat's inferior vena cava using a 5-mL syringe. Alanine transaminase (ALT) and aspartate transaminase (AST) levels were measured to assess liver function.

### 2.4. Preparation and Observation of Liver Tissue Section

The liver tissues were dehydrated using ethanol solution with different concentrations and then embedded in paraffin to create a paraffin block. Tissue sections, 5-*μ*m thick, were sliced from the paraffin block, dewaxed with xylene and ethanol, stained with hematoxylin and eosin (H&E), and then observed under a light microscope.

### 2.5. IL-1*β* and IL-18 in Serum Were Detected by Enzyme-Linked Immunosorbent Assay (ELISA)

Serum levels of IL-1*β* and IL-18 were measured by ELISA kits purchased from Xitang Biological Technology Company. All procedures were performed according to manufacturer's protocol.

### 2.6. Quantitative Real-Time Polymerase Chain Reaction (qRT-PCR) for Caspase-1 and GSDMD in the Liver

Total RNA was extracted using QIAGEN RNeasy Mini Kit (Qiagen, Germany), and cDNA was synthesized with random hexamer primers and RevertAid reverse transcriptase. SYBR Universal PCR Master Mix (Thermo Scientific, United States) was used for amplification. GADPH was used as an internal reference for protein normalization, and the mRNA expression of each group was calculated by the 2^-*ΔΔ*Ct^ method.

### 2.7. Western Blotting (WB)

Fifty milligrams of liver tissue was ground into powder using liquid nitrogen and then lysed by RIPA lysis solution (containing 5% protease inhibitor). The samples were kept on ice for 15 min to ensure complete lyse, centrifuged at 12,000 rpm for 5 min at 4°C, and then the supernatant was collected. The bicinchoninic acid (BCA) method was used to measure protein concentration. After mixing the protein with 5x sodium dodecyl sulfate (SDS) protein buffer, it was denatured in a water bath at 100°C for 10 min. Twenty-five micrograms of the denatured protein was separated, and the protein was transferred to polyvinylidene fluoride (PVDF) by electrophoresis. The PVDF membrane was blocked at room temperature for 2 h, and primary antibody was incubated overnight. Afterward, the membrane was incubated with an horseradish peroxidase (HRP)–conjugated secondary IgG antibody for 2 h at room temperature. Enhanced chemiluminescence (ECL) method was used to visualize protein bands.

### 2.8. Statistical Analysis

Statistical analysis was conducted using SPSS 22.0 software. Measurement data from two independent samples with normal distribution were describe as mean ± standard deviation; data with a skewed distribution were described by median and quartile. For normally distributed data, the homogeneity of variances between two independent samples was assessed using the *t*-test. For data with unequal variances, Welch's *t*-test was used. The Mann–Whitney *U* test was used for data with a skewed distribution. A *p* value of < 0.05 was considered statistically significant.

## 3. Results and Discussion

### 3.1. Animal Models

The rat models of EHS were successfully established. The rectal temperature of the rats exceeded 42.5°C, with an average rectal temperature of 42.6 (42.6°C, 42.8°C). The time required for the rats to reach EHS was 30.7 ± 7.0 min.

### 3.2. Changes of Hepatic Function

The results are represented in Table 1. Serum ALT levels in the EHSG were higher than those in the CG. The AST levels of the EHSG were significantly higher than those in the CG, with a statistically significant difference (*Z* = −2.194, *p* = 0.028).

### 3.3. Significant Pathological Changes Were Observed in the Livers of the Rats in the EHSG

**Figure 1:**
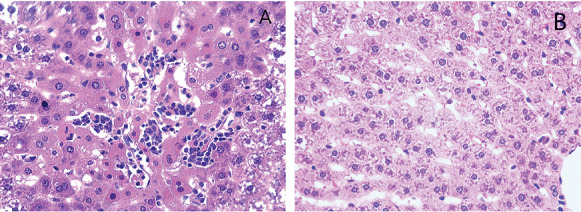
H&E-stained sections of the liver tissue from the two groups of rats. (A) H&E staining of liver tissue in the exertional heat stroke group. (B) H&E staining of liver tissue in the control group. In (A), hepatocytes are markedly swollen and surrounded by a significant inflammatory cell infiltrates (H&E × 200).

Under a light microscope, the liver lobule structures in the CG (Figure 1(B)) were intact, with normal size and morphology of liver cells, and no cell signs of degeneration, necrosis, and inflammatory cell infiltration. In the EHSG (Figure 1(A)), hepatic lobules were disorganized; the hepatic sinusoids were congested; some hepatic cells showed necrosis with pyroptosis characteristics of pyroptosis, such as nuclear pyknosis, fragmentation, and lysis. In addition, inflammatory cell infiltration was observed around the central vein.

### 3.4. Serum Concentrations of Proinflammatory Cytokines Were Significantly Elevated in the EHSG

**Figure 2:**
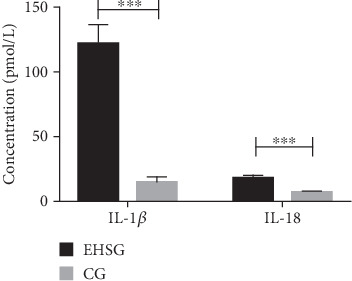
Comparison of serum IL-1*β* and IL-18 levels between the two groups. EHSG represents the exertional heat stroke group. CG indicates the control group. The bar graph demonstrates that serum levels of IL-1*β* and IL-18 levels were significantly higher in the EHSG than in the CG. Data are represented as mean ± SEM for *n* = 12 rats in each group. ⁣^∗∗∗^*p* < 0.001.

As shown in Table 2 and Figure 2, the serum concentration of IL-1*β* in the EHSG was 121.5 ± 51.9 pmol/L, which was significantly increased than in the CG (14.7 ± 11.4 pmol/L) (*p* < 0.001). The serum concentration of IL-18 in the EHSG was 18.0 ± 6.6 pmol/L, which was obviously elevated compared to the CG (6.1 ± 1.8 pmol/L) (*p* < 0.001). These differences are statistically significant.

### 3.5. The mRNA Expression of Pyroptosis-Related Genes Was Significantly Increased in the EHSG

**Figure 3:**
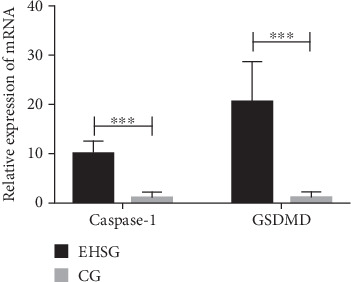
Comparison of mRNA relative expression in liver tissues between two groups. EHSG represent the EHS group. CG indicates the control group. The bar chart shows that the mRNA expression levels of Caspase-1 and GSDMD were significantly higher in the EHSG than in the CG. Data are represented as mean ± SEM for *n* = 12 rats in each group. ⁣^∗∗∗^*p* < 0.001.

As shown in Table 3 and Figure 3, the mRNA relative expression of Caspase-1 in the EHSG was 10.1 ± 2.6, markedly higher than in the CG (1.3 ± 0.9), with a statistically significant difference (*p* < 0.001). The relative expression of GSDMD mRNA in the EHSG was 20.8 ± 8.0, which was significantly higher than in the CG (1.3 ± 1.0), and the difference was statistically significant (*p* < 0.001).

### 3.6. Marked Increase in the Expression of Pyroptosis-Related Proteins Is Observed in EHS Liver Tissues

**Figure 4:**
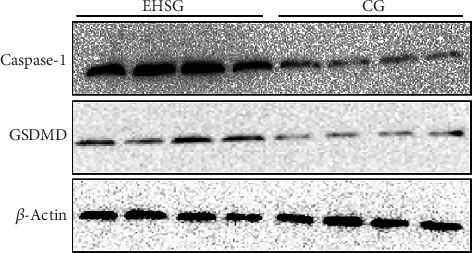
Western blot analysis of liver tissues. EHSG represents the exertional heat stroke group. CG indicates the control group. The first four columns on the left show the protein expressions of Caspase-1 and GSDMD in four randomly selected rats in the EHS group as determined by Western blotting. The four columns on the right correspond to the control group. It illustrates that Caspase-1 and GSDMD expression levels were significantly higher in the EHSG than in the CG.

**Figure 5:**
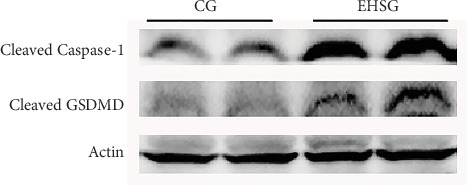
Western blot analysis of liver tissues. EHSG represents the exertional heat stroke group. CG indicates the control group. The cleaved Caspase-1 and cleaved GSDMD in the exertional heat stroke group were significantly increased compared with those in the control group.

As shown in Figure 4 and Table 4, the expression level of Caspase-1 protein in the EHSG increased significantly (5.910 ± 1.620 vs. 1.050 ± 0.360), and the difference was statistically significant compared to the CG (*p* = 0.01). The expression level of GSDMD protein in the EHSG was markedly elevated (0.620 ± 0.12 vs. 0.370 ± 0.020), with a statistically significant difference compared to the CG (*p* = 0.009). In Figure 5, the protein expression level of cleaved Caspase-1 and cleaved GSDMD in the EHSG increased significantly.

With the intensification of the greenhouse effect, global climate is gradually getting warmer, and extreme weather events are becoming more frequent. The incidence of heat stress–related diseases, such as heat spasms, heat exhaustion, and heat stroke, has been steadily increasing, particularly among individuals engaged in production labor, military training, and physical exercise in hot climate, especially in tropical and subtropical regions where the summer temperature and humidity prevail. EHS is a severe illness that poses a significant risk to the health and life of patients. It is prone to cause secondary MODs, including rhabdomyolysis, disseminated intravascular coagulation, and acute liver and renal failure, resulting in a mortality rate of up to 35% [10].

In MODs caused by EHS, liver failure is not only common but also closely linked to the prognosis. Pyroptosis, also known as inflammatory necrosis, is another form of programmed cell death distinct from apoptosis and is mediated by Caspase-1. Pyroptosis is characterized by cytolytic phenomena such as cell swelling and membrane blistering, causing inflammatory responses—changes consistent with the pathological changes in the livers of EHS rats in our previous animal experiments [11]. Other animal experiments have also demonstrated that high levels of IL-1*β* were detected in both plasma and the central nervous system when core body temperature reached 42.5°C, and the trends in core body temperature change were consistent with the plasma IL-1*β* levels in the heat stroke mice [12–14]. These studies all suggest that cytokines play an important role in the occurrence and progression of EHS. IL-1*β* and IL-18 are the characteristic cytokines released in the pyroptosis pathway [15–17]. Therefore, we speculated that pyroptosis may be an important form of injury in the pathophysiology of EHS. To test this hypothesis, we established a rat model of EHS in a stimulated hot climate and conducted an exploratory study. The transcript and protein expression levels of Caspase-1 and GSDMD, key molecules in the pyroptosis pathway, as well as the serum levels of inflammatory cytokines including IL-1*β* and IL-18 in EHS rats were detected in liver tissues. The results showed that Caspase-1 and GSDMD expressions levels were significantly elevated in the liver tissues of EHS rats, and the serum levels of IL-1*β* and IL-18 were also significantly elevated. This finding suggests that the pyroptosis pathways plays an important role in EHS-induced liver injury and that pyroptosis may be one of the important mechanisms of EHS-induced liver injury.

Pyroptosis can be divided into canonical and noncanonical inflammatory pathways. The canonical inflammatory pathway is triggered when pattern recognition receptors (PRRs) detect pathogen-associated molecular patterns (PAMPs) or damage-associated molecular patterns (DAMPs) [18]. This activates the inflammasome, which in turn activates Caspase-1. Activated Caspase-1 cleaves GSDMD, releasing it from its autoinhibition state and forming the active GSDMD-N-terminal. This active form migrates to the plasma membrane and forms a pore approximately10–14 nm in size, leading to increased cell osmotic pressure and rupture due to swelling. At the same time, Caspase-1 promotes the maturation of IL-1*β* and IL-18, which together with other inflammatory factors are released through the GSDMD-N-terminal–formed membrane pores [7, 8, 19]. The noncanonical inflammatory pathway is activated by Gram-negative bacterial endotoxin lipopolysaccharide, which directly cleaves Caspase-4 or Caspase-5 (mediated by Caspase-11 in mice). This activation subsequently leads to the cleavage of GSDMD, further mediating pyroptosis [20].

Caspase-1 is activated from an inactive precursor with size of 45 kDa, which consists of three subunits: an N-terminal subunit of 15 kDa, a central subunit of 20 kDa, and a C-terminal subunit of 10 kDa [21, 22]. The pathogenesis of EHS is generally considered as a sepsis-like syndrome, leading to MOD, a concept accepted widely by scholars. Chen et al. [23] found that during septic acute liver injury, hepatocyte pyroptosis can aggravate acute liver injury and inhibit hepatocyte pyroptosis with NLRP3 and Caspase-1 inhibitors which can reduce the severity of septic liver injury. In our experiment, both the mRNA and protein expressions levels of Caspase-1 in the EHSG were significantly higher than those in the CG.

Numerous studies have identified GSDMD as the executor of pyroptosis [24–26]. Full-length GSDMD consists of an N-terminal domain and a C-terminal domain, connected by a flexible region in the middle [25]. In the resting state, intracellular GSDMD is an autoinhibited state due to the presence of the GSDMD-C terminal. Upon stimulated, activated Caspase-1 or Caspase-4/5/11 cleaves GSDMD, releasing it from this autoinhibitory state [24]. The GSDMD-N-terminal domain (cleaved GSDMD) then combines with phosphatidylinositol and cardiolipin on the cell membrane, forming pores in the plasma membrane [27, 28]. At the same time, GSDMD can further activate the inflammasome and execute the pyroptotic program. Our experiment results showed that the mRNA and protein expression levels of GSDMD in EHS rats were significantly higher than those in the CG. However, some studies have pointed out that the activation of GSDMD is not solely dependent on the cleavage of GSDMD-C-terminus; neutrophil elastase can also hydrolyze GSDMD to produce fragments with pore-forming function that mediate cell death [29, 30]. At the same time, it has been noted that other proteins of the gasdermin family, such as GSDME, can also mediate pyroptosis. Therefore, it is necessary to further explore other molecular mechanisms of GSDMD pore formation in pyroptosis, as well as other proteins involved in mediating pyroptosis.

## 4. Conclusions

The expression levels of Caspase-1 and GSDMD, along with serum IL-1*β* and IL-18, were significantly increased in liver tissue of EHS rats. This suggests that pyroptosis plays an important role in liver injury in EHS rats and may be one of the important mechanisms underlying liver injury in EHS.

## Figures and Tables

**Table 1:** Comparison of hepatic function levels between the two groups.

	**EHSG**	**CG**	**Z** ** value**	**p** ** value**
ALT (U/L)	52.5 (49.5, 62.5)	48.9 (39.6, 54.2)	−2.022	0.045
AST (U/L)	160.5 (124.0, 186.7)	125.4 (116.9, 150.5)	−2.194	0.028

**Table 2:** Serum concentrations of IL-1*β* and IL-18 in both groups.

	**EHSG**	**CG**	**T** ** value**	**p** ** value**
IL-1*β* (pmol/L)	121.5 ± 51.9	14.7 ± 11.4	6.968	< 0.001
IL-18 (pmol/L)	18.0 ± 6.6	6.1 ± 1.8	6.058	< 0.001

**Table 3:** mRNA relative expression of Caspase-1 and GSDMD in both groups.

	**EHSG**	**CG**	**T** ** value**	**p** ** value**
Caspase-1	10.1 ± 2.6	1.3 ± 0.9	10.9	< 0.001
GSDMD	20.8 ± 8.0	1.3 ± 1.0	8.3	< 0.001

**Table 4:** Relative protein expression levels in liver tissues of two groups.

	**EHSG**	**CG**	**T** ** value**	**p** ** value**
Caspase-1	5.91 ± 1.62	1.05 ± 0.36	5.846	0.01
GSDMD	0.62 ± 0.12	0.37 ± 0.02	3.779	0.009

## Data Availability

Data are available on reasonable request. The data that support the findings in our study are available from the corresponding author on request.
